# AGREEMENT BETWEEN CLINICAL AND ANATOMOPATHOLOGICAL DIAGNOSES IN PEDIATRIC INTENSIVE CARE

**DOI:** 10.1590/1984-0462/2021/39/2019263

**Published:** 2021-03-12

**Authors:** Fernanda Staub Rodrigues, Isabella Correa de Oliveira, Mônica Nunes Lima Cat, Maria Clara Lopes Mattos, Gabriela Andrioli Silva

**Affiliations:** a Universidade Federal do Paraná, Curitiba, PR, Brazil.

**Keywords:** Autopsy, Diagnostic errors, Pediatrics, Critical care, Necropsia, Erros de diagnóstico, Pediatria, Cuidados críticos

## Abstract

**Objective:**

Although autopsy is deemed the gold standard for diagnosis, its performance has been decreasing while adverse events have been increasing, of which 17% consist in diagnostic errors. The purpose of this study was to estimate the prevalence of diagnostic errors based on anatomopathological diagnosis in a Pediatric Intensive Care Unit (PICU).

**Methods:**

This is a cross-sectional, retrospective study on 31 patients who died between 2004 and 2014. Diagnoses were compared in order to assess whether there was agreement between clinical major diagnosis (CMD) and the cause of death as described in the autopsy record (CDAR), which were classified according to the Goldman Criteria.

**Results:**

Of 3,117 patients, 263 died (8.4%). Autopsy was conducted in 38 cases (14.4%), and 31 were included in the study. There was a 67% decrease in the number of autopsies over the last 10 years. Absolute agreement between the diagnoses (class V) was observed in 18 cases (58.0%), and disagreement (class I), in 11 (35.4%). There was greater difficulty in diagnosing acute diseases and diseases of rapid fatal evolution such as myocarditis. Seven patients were admitted in critical health conditions and died within the first 24 hours of hospitalization.

**Conclusions:**

Autopsy not only enables to identify diagnostic errors, but also provides the opportunity to learn from mistakes. The results emphasize the relevance of the autopsy examination for diagnostic elucidation and the creation of an information database concerning the main diagnoses of patients who rapidly progress to death in PICU, increasing the index of clinical suspicion of the team working at this unit.

## RESUMO

**Objetivo:**

Embora a necropsia seja considerada o exame padrão-ouro de diagnóstico, observa-se declínio em sua realização, enquanto cresce o número de eventos adversos na saúde, dos quais 17% são erros diagnósticos. O estudo objetivou estimar a prevalência do erro de diagnóstico, com base no diagnóstico anatomopatológico, em uma Unidade de Terapia Intensiva Pediátrica (UTIP).

**Métodos:**

Estudo de corte transversal, retrospectivo, de 31 pacientes que foram a óbito entre 2004 e 2014. Os diagnósticos foram comparados para verificar se houve concordância de diagnóstico principal (CDP) e diagnóstico da causa da morte (DCM), classificados de acordo com os critérios de Goldman.

**Resultados:**

De 3.117 pacientes, 263 foram a óbito. Em 38 casos foi realizada autopsia (14,4%) e 31 foram incluídos no estudo. Observou-se decréscimo de 67% no número de autopsias em dez anos. Concordância absoluta entre os diagnósticos (classe V) foi observada em 18 casos (58,0%) e discordância (classe I), em 11 (35,4%). Observou-se maior dificuldade no diagnóstico de doenças agudas e de evolução fatal rápida, como as miocardites. Sete pacientes foram admitidos em estado geral grave, indo a óbito nas primeiras 24 horas de internação.

**Conclusões:**

A necropsia não só permite a identificação de erros diagnósticos, como também a oportunidade de se aprender com o erro. Os resultados enfatizam a importância desse exame para a elucidação diagnóstica e a construção de uma base de informações sobre os principais diagnósticos envolvidos em pacientes que evoluem rapidamente para o óbito em UTIP, aumentando o grau de suspeição pela equipe da Unidade.

## INTRODUCTION

In 1999, the publication *To Err is Human: Building a Safer Health System*,^1^ of the Institute of Medicine of the United States of America, impacted the population due to the alarming figures presented. The estimate pointed out from 44,000 to 98,000 deaths/year as a result of adverse events of hospitalized patients, with priceless emotional cost and financial cost of 17 to 29 billion dollars.^1^ ^,^ ^2^ Within this context, diagnostic errors are responsible for 17% of cases^2^ and, although this is a worldwide and increasing concern, paradoxically, there is an inverse movement of reduction in the performance of autopsy, an examination recognized by the world's leading health organizations as the gold standard for diagnosis and quality of health care.^2^ ^–^ ^5^

In educational institutions, autopsy is recommended to be performed in at least 25% of hospital deaths.^6^ ^,^ ^7^ Few institutions account for this number, having indices close to or less than 10% of cases.^3^ In this scenario, few doctors receive feedback on the established diagnosis, and pathologists perform less and less the examination during their education, training, and professional life. Although diagnosis procedures have improved in recent decades, the aid of autopsy is invaluable to provide information on the accuracy of diagnoses and answers to open questions, especially regarding the cause of death, being an important tool for the patient's safety systems.^2^ In addition, mortality rates based on autopsies are much more accurate than those based on death certificates, which in turn are based on clinical diagnoses.^8^ ^,^ ^9^

There are many justifications reported for reducing the performance of autopsies, such as costs, cultural, and religious factors.^3^ ^,^ ^8^ ^,^ ^10^ ^–^ ^12^ Although these factors are important to some extent, the main responsible factor for the decrease in the performance of this examination is the argument that advances in technology, associated with the availability of sophisticated methods of investigation, have reinforced the overconfidence and the feeling that autopsy is obsolete and unnecessary.^3^ ^,^ ^5^ ^,^ ^13^ ^,^ ^14^

Diagnostic discrepancies detected by autopsy are typically classified according to the criteria of Goldman et al.^15^ and are based on their clinical relevance and on the potential that timely therapy would have for the final outcome.^4^ They are classified as major (classes I and II) and minor (classes III and IV), and have been used by many authors as a method to evaluate the frequency and impact of diagnoses not determined in life (Chart 1).^15^

**Chart 1:** Goldman et al.^15^ criteria for discrepancies between diagnoses in autopsies.

Classes	Definition
I	The correct diagnosis would have led to changes in conduct, with potential cure or increased survival.
II	The correct diagnosis would probably not lead to changes in conduct and outcome.
III	Diagnostic failure of pathology related to terminal disease, but not related to the cause of death.
IV	Other diagnoses of minor importance ceased to be identified.
V	Absolute agreement.

Source: modified from Goldman et al.^15^.

Although in the literature there are emerging investigations about diagnostic errors in adult patients, with great variation in their rates (5.5–100%),^4^ ^,^ ^10^ relatively speaking, little is known about this topic in pediatrics and even less in Pediatric Intensive Care Unit (PICU) and in Neonatal Intensive Care Unit (NICU).^16^ ^,^ ^17^ In addition, it has been reported that patients admitted to the Intensive Care Unit (ICU) are relatively more likely to suffer damages due to diagnostic errors when compared with those admitted to emergency units or first aid rooms.^4^ Therefore, the objective of this study was to estimate the prevalence of diagnostic discrepancies, based on the anatomopathological diagnosis, in a PICU of a university hospital as well as to classify such discrepancies.

## METHOD

This is a cross-sectional, retrospective study conducted with the analysis of data from patients who died in the PICU of Complexo Hospital de Clínicas of Universidade Federal do Paraná (CHC-UFPR), from 2004 to 2014. Such information, collected from databases of the PICU and the Pathological Anatomy Service, included: age, sex, length of hospital stay, clinical diagnosis, anatomopathological diagnosis, and number of autopsies annually performed. Of 3,117 patients, 263 died (8.4%) and, in 38 cases, autopsy was performed (14.4%), of which 31 were included in the study. Seven cases (22.6%) were excluded because they did not present a conclusive anatomopathological report as for the cause of death.

Cases were studied and clinical and anatomopathological diagnoses were compared to verify if there were agreement between:

- Clinical major diagnosis (CMD) — when the clinical major diagnosis was confirmed by the autopsy;
- Cause of death as described in the autopsy record (CDAR) — when there was a correct diagnosis of the cause of death, confirmed by the autopsy.

Diagnostic discrepancies were classified according to the criteria proposed by Goldman et al.^15^ (Chart 1). Comparisons and classifications were independently carried out by two qualified professionals (professors from the Department of Pediatrics of the CHC-UFPR who agreed to participate in the study), with concordance between both of them regarding all the analyzed cases, considering the objective diagnoses described in the medical records and in the autopsy reports. Furthermore, information about the number of autopsies annually performed was collected, from all deaths that occurred in the CHC-UFPR (and not only cases of the PICU) during the evaluated period. The study was approved by the Ethics Committee of Research involving Human Beings of the institution.

## RESULTS

**Graph 1:**
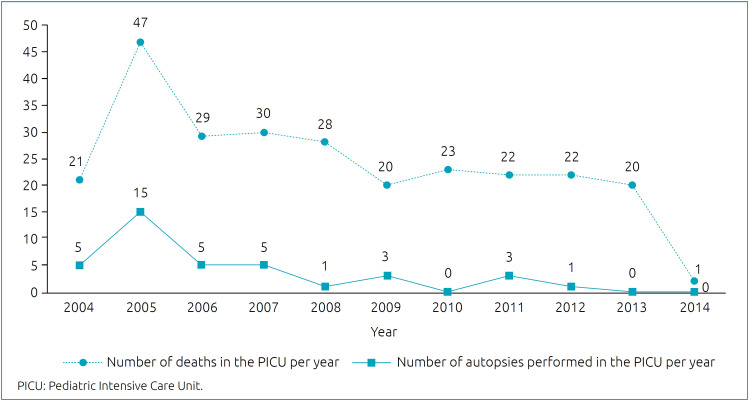
Distribution of the number of deaths and autopsies of the Pediatric Intensive Care Unit per year (2004–2014).

The PICU of CHC/UFPR is a tertiary service unit with eight beds and an average occupancy rate of 110%. From 2004 to 2014, 3,117 patients were admitted and 263 of them died; the autopsy examination was requested in 48 cases, but only performed in 38 of them. Thus, the overall mortality rate in the studied PICU was 8.4% in the ten years, and the autopsy rate was 14.4% (Graph 1).

There was a significant decrease in the total number of neonatal, pediatric, and adult autopsies performed by the Pathological Anatomy Service of CHC-UFPR between 2004 and 2014, with a decrease of 67%. This decrease was substantial between 2007 and 2008 (50.9%), and slower between 2009 and 2014. In 2004, 136 autopsies were performed, and in 2014, 45.

Regarding the clinical characteristics analyzed, it was observed that patients came from the Emergency Care Service (50%), Pediatric Emergencies (24%), Pediatric Infectious Diseases (8%), Pediatric Bone Marrow Transplantation Unit (8.0%), Pediatric Clinics (5%), and Pediatric Surgery (5%), and most of them had clinical diseases, with only two cases of surgical diseases. Thirty-one patients composed the sample of this study, whose distribution per sex was 21 (67.7%) female patients and 10 (32.3%) male patients. The median age was 14 months, ranging from 1 to 156 months, accounting for 3 neonates, 17 infants, 3 preschoolers, 3 schoolchildren, and 5 adolescents. The median length of hospital stay was one day, ranging from 1 to 18 days.

When classified as for the presence or not of diagnostic discrepancy, according to the criteria of Goldman et al.,^15^ absolute agreement between clinical and anatomopathological diagnoses (class V) was observed in 18 (58%) cases. In 11 (35.4%) cases, the clinical major diagnosis and the clinical cause of death were not identified, with potential impact on therapy and the evolution of patients (class I); and in 2 (6.5%) cases, the major diagnosis and cause of death ceased to be identified, but there was no impact on therapy and neither on prognosis (class II). Two cases were classified as classes V and III because, despite the agreement between clinical and anatomopathological diagnoses as for the cause of death, they presented other significant undiagnosed diseases. No case was classified as class IV, i.e., with secondary diagnosis of minor importance (Table 1).

**Table 1:** Characteristics of patients, clinical diagnosis, anatomopathological diagnosis, clinical major diagnosis, cause of death as described in the autopsy record, and classification of Goldman et al.^15^ to assess the agreement of diagnoses of the 31 patients studied.

P	Sex	Ag	LHS	Clinical diagnosis	AP diagnosis	CMD	CDAR	CG
1	F	5	1	Sepsis/BPN	BPN	Yes	Yes	V
2	F	1	1	Gastrointestinal bleeding	Interstitial pneumonia Pulmonary hemorrhage	No	No	I
3	M	1	6	Necrotizing pneumonia	Necrotizing pneumonia	Yes	Yes	V
4	F	6	10	Mitochondriopathy	Mitochondriopathy	Yes	Yes	V
5	F	17	1	BPN	Bilateral lobar pneumonia	Yes	Yes	V
6	M	12	1	Septic shock Meningitis	Septic shock Acute viral myocarditis	Yes	No	I
7	M	53	1	GE/Sepsis	Acute colitis	Yes	Yes	V
8	F	18	1	Acute liver failure	Hepatic cirrhosis/Peritonitis	Yes	No	I
9	F	11	1	Congenital heart disease	Congenital heart disease Interstitial pneumonia	Yes	No	I
10	F	48	1	Acute lymphoblastic leukemia Veno-occlusive syndrome	Acute necrotizing pancreatitis	Yes	No	I
11	F	3	2	Congenital heart disease/BPN	Congenital heart disease/BPN	Yes	Yes	V
12	M	87	17	BMT/Pneumopathy	Pneumopathy/Toxoplasmosis	Yes	No	I
13	F	16	1	BPN	Sepsis/BPN/Purulent cystitis	Yes	Yes	V/III
14	M	5	7	GE	Necrotizing pneumonia	No	No	I
15	F	96	8	BPN	BPN/Myocarditis	Yes	No	I
16	F	32	1	BPN	BPN	Yes	Yes	V
17	M	5	2	Sepsis	Pneumopathy/DIC	Yes	Yes	V
18	F	1	1	Pneumopathy/Sepsis	Pneumopathy/GE	Yes	Yes	V/III
19	F	144	1	Meningitis	Meningitis/Myocarditis	Yes	No	I
20	M	5	1	BPN/Sepsis	BPN	Yes	Yes	V
21	F	3	3	BPN/Sepsis	Sepsis	Yes	Yes	V
22	F	121	1	Meningitis	Meningitis	Yes	Yes	V
23	F	5	2	Myocarditis	Myocarditis	Yes	Yes	V
24	F	14	1	Down/Cardiopathy	Cardiopathy/PH	Yes	Yes	V
25	F	156	1	Severe anemia	Myocarditis/Colitis	No	No	I
26	F	121	1	Meningococcemia	Meningococcemia	Yes	Yes	V
27	F	2	1	Pertussis syndrome	Pneumopathy	Yes	Yes	V
28	F	154	4	Sepsis	Sepsis/Peritonitis	Yes	No	II
29	M	84	18	Hepatic insufficiency	BPN/Hepatic cirrhosis	Yes	No	II
30	M	24	1	Sepsis/BPN	Necrotizing pneumonia	Yes	Yes	V
31	M	12	4	BMT/RPF	Fungal sepsis	No	No	I

P: patient; Ag: age; LHS: length of hospital stay; AP: anatomopathological; CMD: clinical major diagnosis; CDAR: cause of death as described in the autopsy record; CG: Classification of Goldman et al.^15^; F: female; M: male; BPN: bronchopneumonia; GE: gastroenterocolitis; BMT: bone marrow transplant; DIC: disseminated intravascular coagulation; PH: pulmonary hypertension; RPF: idiopathic retroperitoneal fibrosis.

There was CMD, i.e., after performing the autopsy, the major diagnosis surveyed by the clinic had anatomopathological confirmation in 27 (90.3%) out of the 31 cases. The correct CDAR, i.e., cases in which autopsy confirmed the same pathology reported by the clinic as the cause of death, was observed in 18 (58%) patients analyzed.

In the present study, sepsis and bronchopneumonia represented almost all diagnoses of the cases of absolute agreement, with only one case of class I error (undiagnosed necrotizing pneumonia). In the more detailed analysis, a higher frequency of difficulty in diagnosing myocarditis was verified, present in 4 of the 11 cases of class I error.

## DISCUSSION

The results of this study pointed to the same global trend of drastic decline in autopsies (67%), in addition to a high level of disagreement between clinical and anatomopathological diagnoses (58%) and lack of identification of the major diagnosis and clinical cause of death (35%), despite the technological advancement, emphasizing the important role of autopsy in diagnostic elucidation. This examination allows the creation of an information database on the major diagnoses of patients who rapidly progress to death in PICU, thus increasing the index of clinical suspicion of the team working at this unit. Moreover,the examination enables the error to become an opportunity for learning, reflection, and feedback for physicians, contributing to their training and professional growth.

In different epidemiological studies, there has been a decline in the performance of autopsies, either due to medicolegal or didactic purposes.^9^ ^,^ ^18^ Records from the National Center for Health Statistics (United States of America) indicate a decrease from 19.3 to 8.5% between 1972 and 2007.^18^ ^,^ ^19^ In Brazil, according to a study conducted in São Paulo, from 1996 to 1998, autopsy was performed in only 114 (55%) among 206 deaths registered.^19^ The systematic review carried out by Shojania et al.,^20^ which included 53 studies on diagnostic errors related to the major cause of death, presented data on a decline of 30–40% in 1966 to 6% in 1994 (Chart 2). Cuba is the country that still accounts for the highest rates of autopsies performed (55.4%), although it also follows the trend of decrease in the performance of the examination, which results in positive effects on the quality of health care, medical education, research, and innovation.^3^

**Chart 2:** Autopsy rates and discrepancies between clinical and anatomopathological diagnoses (1959–1999).

Period	Local	Patient	Autopsy (%)	Autopsy (n)	Major error (%)	Class I error (%)
1984–1988	Texas University	Adults and children (surgical)	73	409	30.3	7.8
1997–1998	Ryder Trauma Center	Adults and children (traumas)	97	153	15.7	2.6
1984–1993	Lutheran General Children's Hospital	General Pediatrics	36	107	13.1	6.5
1989–1994	University of Rochester	General Pediatrics	74	157	6.4	NA
1992	Children's Hospital of New Jersey	General Pediatrics	29	23	13.0	4.3
1984–1993	Lutheran General Children's Hospital	NICU	61	296	0.3	11.8
1985–1990	Toronto Hospital for Sick Children	NICU	62	338	18.9	2.1
1985–1992	North Shore University Hospital	PICU	26	50	28.0	10
1991–1997	Royal Alexandra Hospital for Children	NICU	40	91	NA	5.5
1995–1996	King Edward Memorial Hospital	NICU	82	197	26.9	12.2
1985–1989	Children's Hospital of Western Ontario	Pediatric Emergency	75	52	15.4	0.0

Source: adapted from Shojania et al.^20^.

NICU: Neonatal Intensive Care Unit; PICU: Pediatric Intensive Care Unit; NA: Data not available.

Of the 53 studies analyzed in the review of Shojania et al.,^20^ only 11 (20.7%) addressed necropsies of neonatal and pediatric patients, which demonstrates the low prevalence of studies on this age group. In the study carried out by Moreira et al.^12^ 160 autopsies performed in the 1970s and 1990s were randomly selected. Of these, 51.8% were of pediatric patients, including perinatal ones, and 48.1% of adult patients, demonstrating a higher rate of autopsy in pediatric patients, possibly because they presented a real clinical challenge due to the wide range of ages, stages of development, and diseases specific to each age group.^12^

The results presented in this study follow the same pattern of the global scenario, considering that, in ten years, there was a 67% decrease in the total number of autopsies performed by the Pathological Anatomy Service. In addition, the same situation can be observed in relation to data specifically concerning the PICU. In 2004, the service presented an annual autopsy rate of 23.8% (5/21), dropping to 0% (0/1) in 2014. At the end of the study, a rate of autopsies performed in the ten years evaluated in the analysis can be estimated: 14.4% (38/263), a percentage far below the minimum recommended for educational institutions (35%).^6^

In this descriptive study, with significantly lower number of cases, the frequency of class I errors was 35.4% (11 out of 31 cases), almost three times higher than the highest rate described in the review conducted by Shojania et al. (12.2%).^20^ This may be related to the low rate of autopsies performed in the study period, indicating a possible bias in the selection of patients submitted to the examination – which is only requested in cases in which there was greater doubt about the diagnosis and difficulty in establishing the etiology of the clinical condition prior to death.

This can be observed in several cases in the present study. Of the 11 cases classified as class I errors, 7 patients were admitted in critical health conditions and died within the first 24 hours of hospitalization, thus hindering the possibility of investigation and the correct diagnosis. This fact demonstrates the greatest difficulty in diagnosing diseases of rapid fatal evolution, such as myocarditis, an etiology of higher prevalence among class I errors in this study.

In order to eliminate the aforementioned selection bias, a Swedish study performed autopsy in 96% of hospital deaths.^21^ The clinical diagnostic error rate of the cause of death was 30%, whereas the diagnostic concordance rate was 57%. This corroborates the hypothesis that autopsies are still necessary to monitor and correct the cause of death, even in cases deemed conclusive,^21^ seeking to maintain the statistically representative autopsy rates of the population.

In the present study, patients were classified according to the presence or absence of CMD and CDAR, aiming at better characterizing the identified errors. In this study, a high rate of CMD (90.3%) was observed. However, there was error in establishing CDAR in 13 (42%) of the 31 cases. In other words, in most cases, the clinical team established the correct major diagnosis, but failed to establish the cause of death of most patients. This demonstrates the importance of autopsy for bringing additional information not only on the major pathological diagnosis, but also on the cause for the patients’ death.

The classification of Goldman et al.^15^ is the criterion used in most studies that compare discrepancies between clinical and anatomopathological diagnoses.^4^ However, it has limitations, for which some modifications have already been proposed in order to better characterize the cases.^22^ Classes I and II, considered as major errors, represent cases in which the main pathology, as well as the cause of death, have ceased to be identified. For class I, the correct diagnosis would lead to a change in the patient's treatment and prognosis. In class II, the correct diagnosis would not cause changes in the conduct and outcome. According to a modification proposed for the criteria of Goldman et al., cases in which there was no timely diagnosis or decision of appropriate therapy should be excluded from class I and, therefore, such cases should be considered as class II errors.^22^ In the present study, according to the modification suggestion, seven cases would be relocated to class II, with a reduction in the rate of class I error from 35.4 to 12.9%. The other classes would have no change in relation to the original criteria.

Diagnostic errors basically have three possible reasons: organizational culture, cognitive process, and poor medical training.^2^ ^,^ ^23^ ^,^ ^24^ Culturally, medical errors or health safety incidents have been addressed with an individual approach, generating guilt, favoring the denial of error, and making it impossible for prevention measures to be effectively implemented. In addition, considering that the health system is typically complex and subject to the interaction of multiple failures, it is a scenario favorable to the occurrence of incidents — the so-called organizational incidents. Thus, underlying problems in the health system also contribute to diagnostic failures and delays. Teams that work poorly together, with poor communication between different professionals, are more prone to diagnostic errors.^2^ ^,^ ^23^

On the other hand, Cognitive Psychology studies how individuals process information. Many diagnostic errors are caused by heuristics or cognitive shortcuts, which are unconscious and automatic processes that guide physicians in the rapid resolution of problems and decision-making, which can assist in clinical reasoning. Although such shortcuts may sometimes be necessary for quick decision-making to save a life, as in many cases in the ICU, they may also predispose the professionals to diagnostic errors in the form of biases.^2^ ^,^ ^23^

There are several cognitive biases that culminate in diagnostic errors, and availability heuristic is one of the most observed biases, occurring when the judgment is made based on the recollection of similar previous cases. Another known bias is that called anchoring or premature closure, when only initial impressions are considered as a basis, disregarding other symptoms that appear later. When the diagnosis is influenced by subtle information, it consists in the framing effect; and, finally, there is the bias of blind obedience, derived from excessive deference to a diagnostic authority or technology.^2^

Concerning errors related to poor medical training, among other issues, the reduction and, often, even the extinction of anatomo-clinical meetings are observed, considered a valuable tool of the medical education.^3^ ^,^ ^11^ Cases of patients who died allow the discussion of the entire clinical history, from the disease presentation to its outcome, the examinations performed, clinical hypotheses elaborated, and therapeutic procedures carried out.^11^ Thus, they enable recognizing that even experienced and well-trained doctors, with access to modern diagnostic resources, are subject to failure in conduct, maintaining the number of incorrect diagnoses still high nowadays.^10^

In addition, the participation in autopsy — previously mandatory during clinical training — was considered a rite of passage, a reference point, a landmark on the path toward becoming a doctor, which brought awareness, morality, and commitment to the profession. This experience, associated with anatomo-clinical meetings and integral cognitive processes, formed the foundation of a good medical training.^25^ It is also noteworthy that health professionals do not receive training during their education on how to approach family members and ask for permission to perform the procedure.^26^ In this context, virtual autopsies — by *post-mortem* imaging (computed tomography or magnetic resonance imaging) — emerge as a complementary tool and can also be used as an option to resume the practice of anatomo-clinical meetings in places or circumstances in which autopsy cannot be performed.^27^

Regarding the limitations of the present study, due to the retrospective design, it was not possible to obtain information about the exact reason for requesting each autopsy examination, mainly due to the lack of data in the medical records. This limitation could be overcome with the development of a prospective study in which all cases of death could be referred to autopsy, thus eliminating the selection bias of more difficult cases, which would make the diagnostic discrepancy rate more representative.
